# Neural systems that facilitate the representation of social rank

**DOI:** 10.1098/rstb.2020.0444

**Published:** 2022-02-28

**Authors:** Madeleine F. Dwortz, James P. Curley, Kay M. Tye, Nancy Padilla-Coreano

**Affiliations:** ^1^ Department of Psychology, University of Texas at Austin, Austin, TX 78712, USA; ^2^ Institute for Neuroscience, University of Texas at Austin, Austin, TX 78712, USA; ^3^ Systems Neuroscience Laboratory, Salk Institute for Biological Studies, La Jolla, CA 92037, USA; ^4^ Department of Neuroscience, University of Florida, Gainesville, FN 32611, USA

**Keywords:** social rank, animal models, neural circuits, social learning

## Abstract

Across species, animals organize into social dominance hierarchies that serve to decrease aggression and facilitate survival of the group. Neuroscientists have adopted several model organisms to study dominance hierarchies in the laboratory setting, including fish, reptiles, rodents and primates. We review recent literature across species that sheds light onto how the brain represents social rank to guide socially appropriate behaviour within a dominance hierarchy. First, we discuss how the brain responds to social status signals. Then, we discuss social approach and avoidance learning mechanisms that we propose could drive rank-appropriate behaviour. Lastly, we discuss how the brain represents memories of individuals (social memory) and how this may support the maintenance of unique individual relationships within a social group.

This article is part of the theme issue ‘The centennial of the pecking order: current state and future prospects for the study of dominance hierarchies’.

## 1. Introduction

Dominance hierarchies are an important form of social organization found in numerous social species, yet little is known about the neural mechanisms facilitating the establishment of social rank. While the neural mechanisms of aggression—an important feature of dominance—have been explored and reviewed elsewhere [1,2], less is known as to how social rank is represented in the brain.

**Figure 1.**
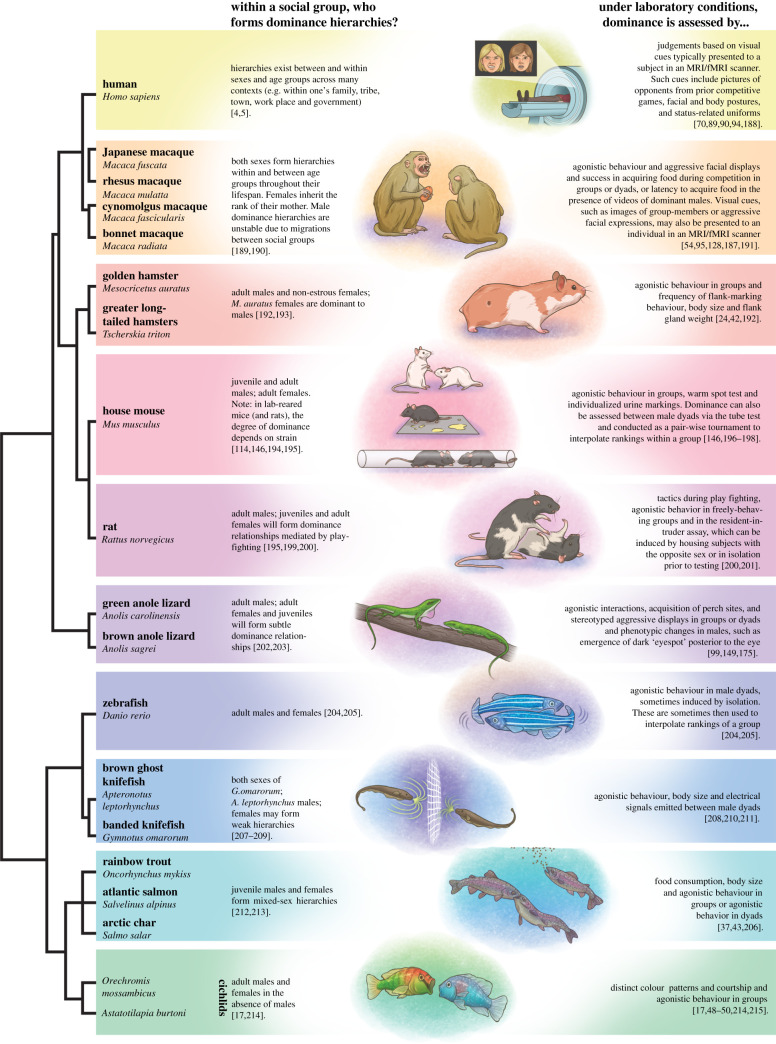
Common species used to study social dominance in the laboratory. Fish, lizards, rodents and primates have been used to study the neural basis of dominant and subordinate behaviour in the laboratory setting. On the left, the life stage and sex in which hierarchies have been documented are listed. On the right, how dominance is assessed in the laboratory conditions is noted. Figure art by Amy Cao.

There is extensive behavioural evidence that animals are aware of their rank and the ranks of other group members. For example, *attention hierarchies*, or the monitoring of more dominant individuals by subordinates, have been observed in species ranging from humans to fish to facilitate avoidance of aggression, recognition of opportunities to rise in rank, and observational learning from successful individuals [3–9]. Importantly, in many species social ranks are not inherited and animals establish social ranks via social experience, suggesting learning mechanisms are necessary for social hierarchy formation [10–13]. An animal's ability to evaluate the social rank of nearby conspecifics is a crucial first step in the contextually appropriate expression of dominant or subordinate behaviour, and in turn the maintenance of stable social ranks. In this review, we explore the neural circuits supporting the evaluation and comprehension of social rank among social species ranging from fish, reptiles, rodents and primates (figure 1), with emphasis on mechanisms with conserved function across these taxa. We first examine hypothalamic, mesolimbic and cortical circuits involved in the perception of status signals used to rapidly assess rank in a generalizable manner. We then present evidence that signal-detecting brain regions overlap with those involved in more general social learning processes and speculate as to how they may support the learning of social rank relationships, a relatively understudied process. Lastly, we explore evidence for individual-based recognition of social rank and the mechanisms that may support more fine-tuned and cognitively complex representations of social relationships among group-living species.

## 2. Representation of status signals

**Figure 2.**
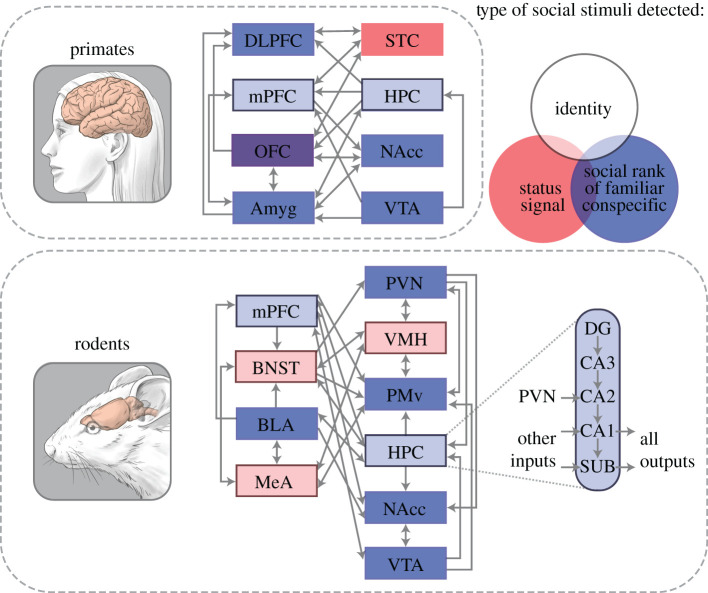
Brain systems involved in the representation of a dominance hierarchy. Brain regions downstream of primary sensory processing regions (e.g. olfactory regions) involved in representing social rank are colour coded by whether local activity has been shown to represent status signals, social rank of familiar conspecifics, and social identity, as well as any combination of those types of conspecific stimuli in primates (top) and rodents (bottom). Hippocampal subregions in the rodent brain and their input and output locations are displayed in the blow-out diagram. Grey lines represent anatomical connectivity across regions. CA1, cornu ammonis 1; CA2, cornu ammonis 2; CA3, cornu ammonis 3; DG, dentate gyrus; DLPFC, dorsolateral prefrontal cortex; mPFC, medial prefrontal cortex; OFC, orbitofrontal cortex; Amyg, amygdala; STC, superior temporal cortex; HPC, hippocampus; NAcc, nucleus accumbens; VTA, ventral tegmental area; BNST, bed nuclei terminalis; BLA, basolateral amygdala; MeA, medial amygdala; PVN paraventricular hypothalamus; SUB, subiculum; VMH, ventromedial hypothalamus; PMv, ventral premammillary nucleus.

The expression of dominant or subordinate behaviour does not always require physical competition, but rather can be based on conspecific cues about individuals' competitive ability known, as status signals (see Tibbetts *et al*. [14]). It has been theorized that status signals evolved to convey crucial information between conspecifics, such that these signals provide a dominant individual with priority access to territory, resources and mates while also facilitating avoidance of aggression by subordinate individuals [8,15–17]. These signals are often associated with a cost to the signaller, such as androgen production or an increased risk of predation to reinforce their honesty [16]. Importantly, status signals are not any social stimuli of a given conspecific of known social rank, rather they are species-specific features that convey competitive ability regardless of familiarity with conspecifics. Status signals have been extensively documented in a variety of species, come in a wide variety of forms ranging from pheromones to complex behavioural displays, and often signallers adopt more than one signalling modality. Thus, the perception of social status is usually more complex than detecting a single signal and involves the integration of multiple features [18]. Although there is limited understanding of how the brain processes status signals, several brain regions and circuits have been implicated in processing status signals through experiments involving presentations of chemical and visual status signals (figure 2). Interestingly, several of these brain regions are also relevant in general avoidance and reward learning and memory processes, thus we also speculate as to whether the representation of status signals is innate or learned through prior social experiences.

### (a) Insights from chemical signalling

#### (i) Extended amygdala and hypothalamic circuits process chemical signals in rodents

Chemical signals, such as pheromones and urinary proteins, are perhaps the most common mode of communication used by social species to convey identifying information including sex, species, status and individual identity [19–23]. The use of chemical signals may be especially important in territorial species, such a rodents [20,24]. Neural circuits that detect social odours are well characterized in small mammals that use both main olfactory (volatile odours) and vomeronasal/accessory (non-volatile odours) systems. The vomeronasal organ (VNO) neurons project primarily to regions implicated in social behaviour and the canonical view is that the vomeronasal system is specialized to detect species-specific chemical signals that carry information about sex, reproductive or dominance status, but there is some evidence that the main olfactory system also processes social chemicals [25]. In male mice (*Mus musculus*), specific receptors in the VNO have been identified as necessary for sex cue discrimination and expression of sexual and aggressive behaviour [26,27]. In both female and male mice, downstream targets of the VNO exhibit greater activation upon sniffing urine from dominant males compared to urine from subordinate males, as evidenced by number of *cfos* immunoreactive cells, a proxy of neuronal excitation [28,29]. This activity could represent the detection of higher levels of major urinary proteins (MUPs), some of which are crucial for territory marking [30]. Furthermore, in rodents specific MUPs can signal dominance [31,32].

One of these downstream regions is the medial amygdala (MeA), which receives input from both vomeronasal and main olfactory systems and is an early node in the odour processing stream across vertebrates [33–35]. The MeA is also a highly sexually dimorphic nucleus rich in sex steroid hormone receptors [36] and has been implicated in sexual and aggressive behaviours in rodents, fish and lizards [37,38]. Sexual dimorphisms also appear to influence how the MeA represents social information. Calcium imaging in the MeA of male and female mice revealed that Ca^2+^ dynamics of neuronal ensembles and individual neurons differentiate between conspecific cues in a sex-specific manner, suggesting that biological relevance shapes social odour representations in the MeA [39]. Furthermore, the neuropeptide oxytocin (OT), which is known to mediate a variety of social behaviours, facilitates the MeA's role in sexual recognition as male animals become sexually mature, suggesting that OT in the MeA serves to encode biologically relevant odours [39–41]. How OT modulates social status signal processing in MeA remains unknown, but we hypothesize that OT facilitates the encoding of relevant social status odour cues in MeA. Interestingly, in male mice, the MeA has more neurons with *cfos* immunoreactivity (*cfos*-ir) after subjects are exposed to urine from an alpha (i.e. the most dominant individual in a hierarchy) compared to a subordinate conspecific, and this does not depend on the subject's own social rank or their familiarity with the cue source, although these factors may influence the biological relevance of the cue [29]. Activity in the MeA is also increased in both dominant and subordinate greater long-tailed hamsters (*Tscheskia triton*) following agonistic encounters and is not correlated to expression of aggression or defensive behaviour [42]. These findings may suggest that activity in this region represents sensory input or arousal in a general manner and that it may not be crucial for the expression of contextually appropriate dominant or subordinate behaviour. However, there is some evidence that the role of the MeA in chemosensory processing is more fine-tuned, as OT transmission in the MeA may be required for encoding memories of individual conspecifics (see §4b).

While the MeA plays a critical role in identifying social rank based on olfactory cues from mouse urine, it is not clear whether this process is further modified by social memory. Interestingly, several MeA hypothalamic projection targets are activated upon exposure to urine and are modulated by social rank and familiarity with the urine source [29]. For example, in the most subordinate mice from a hierarchy, differential *cfos*-ir within the ventromedial hypothalamic nucleus (VMH) is greater when they are exposed to alpha versus subordinate urine regardless of whether the urine is from a familiar mouse. However, in the most dominant (alpha) mice from a hierarchy, *cfos*-ir in the VMH is only greater when they are exposed to *unfamiliar* alpha urine compared to either familiar or unfamiliar subordinate urine [29]. Thus, *cfos*-ir in the VMH upon exposure to urinary cues is modulated by familiarity with the cue, the social rank of the animal who provided the cue, and the animal processing the cue. How the social rank of an animal modulates how it processes social cues is expanded upon in §2a(iii). Also, in both dominant and subordinate animals, the ventral premammillary nucleus (PMv) exhibits greater *cfos*-ir when animals are exposed to familiar alpha urine compared to familiar subordinate urine. Interestingly, this differential *cfos*-ir in the PMv is not observed when animals are exposed to unfamiliar alpha versus unfamiliar subordinate urine. This suggests that the PMv is not detecting features of urine cues that would necessarily qualify as status signals and that are related objectively to social rank regardless of familiarity. Rather, the PMv appears to detect features that are associated with memory of social rank.

This evidence from chemical processing of social odours in rodents suggests that various brain regions represent social rank at different scales. Some regions, such as the MeA, appear to detect status signals, as they exhibit differential *cfos*-ir in response to dominant versus subordinate cues regardless of familiarity with a cue. Others, such as the VMH and PMv, are modulated by additional factors, including an animal's own social rank and familiarity with the individual providing the cue, suggesting that features of learned social rank relationships or social memory are also represented in these regions.

#### (ii) Chemical status signalling in other species

The neural circuits for processing social chemosensory cues are less well characterized outside of rodents, although social chemicals appear to be especially important in other taxa living in environments where vision is obscured, such as nocturnal primates and aquatic animals living in turbid environments [43,44]. Like rodents, dominant male cichlid fish (*Astatotilapia burtoni* and *Oreochromis* spp.) use urine to signal dominant status and have increased urine storage compared to subordinates [45,46]. When tank water is renewed and secreted chemicals are thus removed, pairs of fish are unable to form stable dominance relationships due to contextually inappropriate expression of aggression by subordinates towards dominant individuals [47]. Interestingly, when dominant male cichlids are exposed to a chemical status signal from another dominant male, they increase urination frequency and levels of circulating androgens and gene expression patterns in olfactory processing regions are associated with exposure to various urinary cues [48,49]. Dominant males also exhibit differential gene expression profiles in the olfactory bulb (OB) and posterior portion of the dorsal telencephalon (Dp; the putative homologue of the mammalian olfactory cortex) when they are exposed to dominant versus subordinate urinary odours [50]. Furthermore, neural recordings from the ventral nucleus of the fish ventral telencephalon (Vv; homologous to the lateral septum (LS) and striatal external globus pallidus of mammals) indicate that dominant males more robustly differentiate between sex- and food-related odours in these regions, while brain activity in subordinates only distinguished the odour of dominant males [50]. Thus, the social rank of fish exposed to social cues is a factor that influences how they process these cues, as is also observed in mice (see §2a(i)).

Unlike fish and rodents, primates are generally assumed to rely on visual information rather than chemical or olfactory information when assessing social cues [51]. However, behavioural studies in nocturnal New World monkeys (e.g. Lemuridae, Indridae, Callitrichidae and Cebidae) indicate that biological odours may be involved in activities related to dominance, such as territorial marking and reproductive behaviour [52]. Research into how the primate brain processes social olfactory cues is limited; however, studies have shown that the size of the accessory olfactory bulb (AOB) correlates with social and mating systems such that species with more dispersed social networks have greater AOB volume [53], suggesting that olfactory pathways similar to those in rodents are used to process status signals in nocturnal New World monkeys. More research is needed to determine whether primates detect social rank from chemical cues. Currently, the majority of what is known about how the primate brain represents status signals stems from experiments using visual stimuli.

### (b) Insights from visual signal processing

#### (i) Amygdala processing of visual status signals in primates

Some of the earliest investigations into the neural correlates of dominance hierarchies identified the amygdala as a crucial brain region for appropriate expression of dominant and subordinate behaviour. For example, bilateral amygdala lesions given to the highest-ranking macaques (*Macaca* spp.) in a dominance hierarchy resulted in drastic but variable effects on dominant and subordinate behaviour and social rank changes. This included a complete reduction in aggression and loss of high status in one individual, as well as increase in aggression and a transition from typical dominant behaviour to despotic dominance in another individual [54]. Human lesion studies, such as those characterizing Klüver–Bucy syndrome [55], further support the amygdala's broad role in contextually appropriate social behaviour. Furthermore, individuals' social network complexity correlates with amygdala volume in human and non-human primates, suggesting that the amygdala serves a role in navigating the social environment [56,57].

For decades we have known that the amygdala is critical for encoding and signalling the affective valence of stimuli, including social stimuli [58–62]. Electrophysiological and neuroimaging studies have shown that the amygdala increases activity when macaques and humans view facial expressions of unfamiliar individuals who are angry or threatening [63–65]. In humans and monkeys, angry facial expressions and direct eye contact in images of single individuals are associated with dominance, as this indicates aggression directed toward the perceiver [64,66]. Conversely, fearful faces in macaques have been associated with submission, or a lack of aggression [64]. Interestingly, in humans the evoked blood-oxygen-level dependent (BOLD) signal response in the amygdala is even greater in response to fearful faces, suggesting that not knowing the source of a threat activates the amygdala more strongly [65,67]. This supports the idea that that a major role of the amygdala is to acquire salient social information in the environment.

#### (ii) Amygdala's potential role in learning about social rank stimuli

The degree to which animals innately recognize status signals or must learn to appropriately interpret them is not known. Typically, the process of signal detection and response is presented as innate; however, this may not be true for all types of signals. For instance, amygdala lesions in neonatal monkeys increase social fear in social interactions despite a lack of warning facial expressions [68], but lesions in adult monkeys do not have this effect and instead lead to a reduction of cautious behaviours towards unfamiliar individuals [69]. These studies suggest that a period of socialization and learning is necessary to effectively interpret conspecific behaviour and signalling [69]. Furthermore, in the absence of status signals, activity in the amygdala is associated with social ranks of familiar individuals. For example, neuroimaging studies in humans show that BOLD signal in the amygdala increases when subjects view neutral facial expressions of familiar dominant competitors, and in macaques basolateral amygdala (BLA) firing rate correlates with social ranks of neutral familiar faces [70,71]. This suggests that the amygdala may also represent learned social rank or emotional memories associated with dominant individuals.

The role of the amygdala in fear and threat learning associations has been thoroughly studied in rodents in the context of Pavlovian conditioning. While conditioning experiments have traditionally demonstrated the learning of associations between an electric shock and an object or environmental context, it is likely that physical injury incurred through competition is associated with status signals in a similar manner. Briefly, the circuitry underlying the fear conditioning process involves the BLA, which integrates sensory information about the external environment and an animal's internal state with contextual information and memories from the hippocampus (HPC) and prefrontal cortex (PFC) [72,73]. This is where associations between fear-evoking stimuli (i.e. unconditioned stimulus; US) and neutral stimuli (i.e. conditioned stimulus; CS) are learned, and then this information is transferred to the central amygdala, which projects to midbrain regions involved in behavioural output [74]. Interestingly, this circuitry is engaged when rodents learn fearful associations directly and when they learn vicariously by observing conspecifics (i.e. vicarious learning), with additional top–down input from the PFC for sustaining attention during vicarious learning [75]. Subordinate animals may use vicarious learning via observations of agonistic interactions between group members to determine social ranks and avoid potential injuries associated with direct learning. While associative learning mechanisms that link the competitive ability of conspecifics to status signals could underlie the representation of status signals in the amygdala, this has not been empirically tested. Furthermore, the responsivity of amygdala sub-nuclei (i.e. BLA) to status signals could be further resolved to substantiate or disprove this theory. In addition, these mechanisms could also facilitate learning associations between competitive ability and individual conspecifics, rather than generalizable signals, and this will be explored further in §3a.

#### (iii) Dopaminergic signalling and ventral striatum responsivity to visual status signals

Across species, the dopaminergic system is involved in processing social status signals. In cichlid fish, presentation of a dominant male is associated with increased number of dopamine (DA) neurons with *c-fos* immunoreactivity in the central region of the ventral telencephalon (Vc), a homologue of the mammalian striatum [48]. In green anole lizards (*Anolis carolinensis*), DA signalling in specific brain regions is elevated in various brain regions in response to changes in status signals. Males that viewed an opponent with a covered dominance-signalling eyespot had increased DA in the ventral tegmental area (VTA), substantia nigra (SNR), nucleus accumbens (NAcc) and hypothalamus, and became dominant. By contrast, males that viewed opponents with artificial eye-spots had increased DA in the dorsal raphe nucleus (DRN) and amygdala, and became subordinate [76].

Lesioning regions that are strongly innervated by dopaminergic neurons also appears to impair status signal detection. Lizards with unilateral lesions to the ventral striatum are unable to detect dominance signals from intruder conspecifics presented to the part of the visual field corresponding to the lesioned hemisphere, but no impairment is seen when they are presented to the intact hemisphere [77]. Interestingly, damage to the ventral striatum in humans (*Homo sapiens*) also impairs the ability to recognize angry unfamiliar facial expressions [78].

Beyond signal detection, the release of DA serves as a prediction-error signal alerting discrepancies in the experienced value of a cue and its expected value. This phenomenon builds on the Pavlovian learning system, such that DA signalling can assign strength and valence to CS–US associations and facilitate reinforcement learning of action patterns such as social approach and avoidance [79,80]. Similar to the representation of status signals in the amygdala, it is unclear whether dopaminergic signalling in response to status signals is innate or learned through prior social experience. There is evidence from primate studies that increased DA signalling in response to dominant stimuli is a learned response. For example, neuroimaging in humans has shown that the ventromedial striatum, which receives information about reward from dopaminergic input, exhibits greater BOLD signal when subjects are presented with the faces of familiar dominant individuals with neutral facial expressions [70]. Electrophysiological recordings in macaques also reveal a population of neurons in the medial striatum that specifically signal social information that is enhanced when viewing dominant individuals [81]. Furthermore, male macaques will sacrifice juice rewards for the opportunity to view the faces of familiar high-status monkeys [82]. Although these studies do not record dopamine signalling directly, these findings suggest that elevated DA signalling may reflect the greater value of viewing dominant group members, as these provide salient threat information. Future studies using the recently developed dopamine imaging sensors could address this hypothesis [83].

#### (iv) Cortical processing of visual status signals in primates

Considering the prominent role of the PFC in social cognition and in decision making [84–86], it is not surprising that multiple prefrontal regions respond to social status signals. For instance, the orbitofrontal cortex (OFC), exhibits greater BOLD signal in response to images of conspecifics compared to non-social stimuli. Furthermore, OFC firing rate differentiates between images of familiar dominant and subordinate conspecifics in rhesus monkeys [87]. Activity within the OFC is also correlated with social image value as measured by time spent looking at the images [87]. However, it is unclear if OFC represents learned status recognition or some dominance-related facial feature, as the individuals in this study were exposed to familiar faces only. Studies in humans have shown that the OFC is important for recognizing emotion in facial expressions and voices from unfamiliar subjects [88]. Furthermore, neuroimaging studies have shown that the dorsolateral prefrontal cortex (DLPFC) also exhibits greater BOLD signal to unfamiliar individuals in postures associated with high status [89–91], and interestingly, to social status of neutral faces of contest opponents [70].

Prefrontal representations of status could also reflect attentional processes and the salience of viewing a dominant individual. This could explain why similar BOLD signal responses are observed when subjects are presented with dominant facial expressions and neutral faces of familiar dominant individuals. The PFC's role in sustaining attention has been extensively studied [92]. The elevated PFC activity observed in response to dominant signals could indicate that status signals serve to direct the attention of animals in a group towards dominant focal individuals, and this could manifest as an attention hierarchy. Interestingly, human participants who perceive themselves as low-status are more sensitive to facial dominance signals [93]. This potential skew in PFC-mediated attention could facilitate the acquisition of salient social information, i.e. which group member(s) are highly combative.

Beyond the PFC, parietal cortical regions are also involved processing visual status signals. For example, the superior temporal cortex (STC) exhibits greater BOLD signal when human subjects are presented with dominant facial features and postures and when they use these features to make judgements regarding the relative dominance of two other individuals [89,94,95]. There is also evidence to support that the STC works in conjunction with the inferior temporal gyrus, or fusiform gyrus, and HPC to compile information about emotions and intentions reflected in faces and the individual identity of faces [96–98]. Representations of individual social memories will be further explored in §4a.

## 3. Representation of learned social rank in approach-avoidance circuits

Status signalling is not the only mode by which animals perceive social rank. Even species that rely on status signals appear to prioritize behavioural demonstrations of dominance in competitive interactions and memories of those interactions over status signals to guide their behavioural decisions. For example, in lizards, manipulating the dominance-signalling dark eyespot does not alter previously established dominant–subordinate relationships [99], but it does determine dominant–subordinate relationships between unfamiliar dyads [76]. Furthermore, status signals do not explain how animals are able to detect subtle rank gradations that exist in highly linear dominance hierarchies where individuals behave differentially towards their two closest-ranking group members. For instance, there are no apparent status-driven differences in the urinary odour profiles of lower-ranking mice from the same hierarchy [31]. Here, we argue that animals must also learn social ranks and how to behave appropriately with specific group members through direct experiences and interactions with competitors, observation of contests between other individuals (*observational* or *vicarious learning*), or the determination of social status based on cognitive processes such as transitive inference [12].

Notably, these learning processes and status signalling are not mutually exclusive and coexist. As mentioned in §2b(iv), elaborate status signals may serve to direct attention towards dominant conspecifics, which is crucial for learning about them. This could manifest as an attention hierarchy, which then facilitates learning about and from highly ranked group members [3]. Furthermore, a stressful event, such as presentation of a dominant signal, can elevate levels of stress hormones and neuromodulators that enhance learning and memory [100]. Stress reactivity and the neuroendocrine characteristics associated with status signalling and social rank are too exhaustive to discuss in this review (see Milewski *et al*. [101]). In the following sections, we discuss neural mechanisms that we speculate support associative learning between competitive ability and individuals, rather than a generalizable signal.

### (a) Amygdala circuits facilitate learning of social subordination and vigilance

We propose that the amygdala facilitates learning about social rank relationships through competitive social interactions. With the establishment of dominance relationships, subordinate animals yield to higher-ranking group members and avoid pain and injury incurred by fighting [72], a phenomenon that likely involves fear and threat learning systems. Studies in rodents using social defeat stress (SDS) paradigms may support this theory, with some caveats. During SDS, animals are continually subjected to agonistic interactions and the primary measure of successful conditioned social defeat is the animal's decreased propensity for social interaction. SDS studies have illustrated that similar neural correlates in the amygdala underlie social submission and fear learning. For example, brain derived neurotrophic factor (BDNF)-mediated plasticity and GABAergic transmission within the BLA are critical for acquisition and expression of conditioned fear as well as conditioned social defeat in rodents [102–105]. Projections to the amygdala from the medial PFC (mPFC) have also been shown to similarly modulate fear conditioning and responses to SDS [106,107]. The role of BLA homologues has also been explored in non-mammals. In ray-finned fish, the medial dorsal telencephalon (Dm) is believed to be homologous to the BLA. The Dm has been implicated in emotional learning and fear avoidance [34,108,109], and local activity measured by number of *cfos* immunoreactive cells increases over subsequent days of SDS [110], suggesting that it also mediates learning of social defeat and development of social avoidance.

While amygdala circuits involved in fear and social avoidance may underlie how the most low-ranking animals in a group may learn their subordinate status, it does not explain how intermediately ranked animals learn to flexibly avoid and submit to some group members and exhibit aggression towards others. Generalized social avoidance induced by fear learning of conspecifics or conditioned social defeat would prevent intermediate animals from expressing both submissive and dominant behaviour when appropriate and prevent socially enriching affiliative behaviours. Another possibility to explain flexible behaviour in intermediates is that social defeat in naturalistic settings induces an anxiety-like social state, such as social vigilance, rather than complete avoidance. Social vigilance, or monitoring of conspecifics, could enhance observational learning and selective avoidance of aggressive conspecifics. While hypervigilance is considered a maladaptive response to uncertainty and associated with higher risk for anxiety disorders [111], an appropriate degree of vigilance is considered an adaptive coping mechanism adopted by lower-ranking animals to avoid potential threats and seize opportunities to increase their rank or acquire resources [112]. Studies in mammals show that the bed nucleus of the stria terminalis (BNST), a component of the extended amygdala, signals valence of stimuli in ambiguous social contexts and thus mediates social anxiety and social vigilance [113]. In humans, activity of the BNST is greater than activity in the amygdala when participants are exposed to unpredictable associations between cues and threatening facial expressions [111].

Social vigilance also appears to be modulated by one's own social rank. In primates, rodents and fish, social vigilance is skewed, giving rise to attention hierarchies where subordinates have elevated levels of social vigilance [3–9]. In many species there is a negative association between stress hormone levels and social rank, although high levels have also been observed in dominant individuals, revealing U-shaped association between stress hormones and social rank [43,114–117]. Most studies have focused on stress responsivity to antagonistic social interactions in subordinates or socially defeated animals. Following SDS in mice, release of the stress-related neuropeptide corticotropin-releasing factor (CRF) from the paraventricular nucleus (PVN) is upregulated [118]. Interestingly, CRF antagonism specifically in the BNST blocks the conditioned social defeat in hamsters [119]. These findings support a model in which stress hormones mediate processes in the BNST required for social defeat learning.

In addition to stress hormones, OT signalling varies based on an individual's social rank and mediates social vigilance. In female macaques exposed to stress, dominant social status is associated with elevated OT in cerebrospinal fluid [120]. In male rats, OT-receptor (OTR) binding is greater in the BNST and central amygdala (CeA) in more aggressive strains [121], and in male mice, OTR binding is associated with dominant social rank in other extended amygdala nuclei such as the NAcc and LS [122]. Interestingly, OTR knockdown in the BNST of male mice prevents social stress-induced increases in social vigilance and decreases in social approach [31]. In male macaques, pharmacological delivery of OT leads to increased prosocial behaviour and decreased subject vigilance for viewing dominant faces [123]. Collectively, these data suggest that mechanisms within the BNST and social rank-associated differences in stress hormone and OT levels could drive social vigilance in low-ranking animals, and in turn, the formation of attention hierarchies.

While the function of the BNST in relation to social behaviour has not been thoroughly studied in non-mammalian species, recent evidence from fish indicates that activity in the supracommissural subdivision of the ventral telencephalon (Vs), considered homologous to a combined MeA/BNST complex, is increased during cue learning in social (i.e. group-living) compared to non-social conditions [124]. Thus, the reactivity of the BNST in social situations appears to be evolutionarily conserved.

### (b) Mesolimbic dopamine system mediates social dominance and subordination behaviours

The dopaminergic system is another associative learning system that may be involved in social rank learning. As described in §2b(ii), elevated DA signalling is observed when animals are presented with dominant signals. Further, dopamine neurons in the DRN modulate social behaviour in a social rank-dependent manner, with the impact of activating DRN DA neurons greater in more dominant mice [125,126]. Although it is not known whether this is a result of social learning processes or an innate response to the signal, there is evidence to suggest that DA signalling may be involved in learning social rank through social interactions in the absence of signals.

Studies in rodents have shown that reward learning via dopaminergic signalling mediates aggressive behaviour. In dominant animals, aggression serves as a naturally rewarding US and can reinforce behaviours that lead to attacking a submissive animal [127]. In both macaques and mice, dominant males exhibit greater binding of the D2-type DA receptor in the striatum and D2 antagonism attenuates aggression [128]. In macaques, D2 antagonism results in destabilization of dominance hierarchies when specifically administered to the dominant animals and not subordinates [128,129]. Some studies have also shown that D1 and D2 receptor antagonism can attenuate aggression in rats and mice [130–133].

While not directly tested in a dominance hierarchy context, a highly conserved dopaminergic circuit has been identified in rodents and primates (figure 2), in which dopaminergic neurons from the VTA to the NAcc (part of the ventral striatum) facilitate learning of social reward and promote social competitiveness [132,134,135]. It should be noted that other neuropeptides, serotonin and OT, are also important for social reward learning within this circuit in mice [59,60]. Similar projections have been identified between VTA and NAcc homologues in birds, reptiles, amphibians and fish that motivate social approach in mating and courtship behaviours [136–140].

It is not known whether differences in DA receptor expression pre-determine an animal's social rank or whether they are the result of acquired social rank differences. It is possible that DA signalling differences are inherent as well as mediated by experience, such that animals with initially greater motivation experience greater reinforcement of this behaviour through the rewarding aspects of winning. Elevated DA signalling has been associated with the winner effect, a phenomenon where winning fights perpetuates further winning against new individuals in the future, leading to increased social status [133,141]. Furthermore, humans highly motivated to win competitions exhibit greater ventral striatal activation when viewing images of opponents [70,142].

Interestingly, DA signalling is also involved in mediating subordinate behaviour. As discussed in §3a, SDS is similar to the repeated defeat experienced by subordinate animals in a hierarchy. DA transmission in the VTA–NAcc circuit has been implicated in social defeat learning in mice and hamsters by modulating plasticity [143,144]. Furthermore, in socially monogamous rodents, SDS induces an upregulation of D1 receptors in the MeA, an important region for processing of social information [145]. Thus, changes in DA signalling following SDS suggest that individual differences in DA transmission and DA receptor expression may be a result of acquiring a given social rank and not a pre-existing correlate of social rank. These studies also suggest that the rewarding versus aversive effects of DA signalling are brain-region specific.

In summary, dopamine circuits across species facilitate social reward learning and mediate an animal's decision to engage with or avoid certain individuals. This learning mechanism could be used as animals develop social hierarchies and learn their social ranks within that hierarchy.

## 4. Individual social memory

Animals living in social dominance hierarchies have to behave dynamically, such that they express dominant behaviour towards some individuals and subordinate behaviour towards others. Therefore, social memory of individual group members is required in conjunction with the associative learning processes that facilitate approach and avoidant social behaviours. In highly linear dominance hierarchies, where each individual attains a unique social rank, animals behave based on subtle rank differences. For example, dominant male mice tend to exhibit the majority of aggression towards closely ranked competitors [146], and male chimpanzees tend to groom only closely ranked males [147]. This socially selective behaviour suggests that animals have strong individual social memories of their conspecifics. Whether animals always rely on individual social memories or use generalizable features associated with status (i.e. status signals) may depend on dynamics of the social environment and selective pressures. For example, male cichlids living in highly dynamic social environments form short-term memories of dominant rivals and will only express submissive behaviour towards males with dominant morphology who have demonstrated superior fighting ability within the past 7 days [148]. Similarly, lizards form social memories from a single social interaction, but the salience of this social identity information appears to decline and is eventually forgotten as intervals between subsequent interactions increase [149]. In addition, group-housed but not single-housed mice demonstrate long-term memory of individuals even after very brief interactions, further supporting the idea that social memory is dependent on social context [150]. Below we review the neural basis of individual social recognition that may facilitate learning of individual social ranks in a dominance hierarchy.

### (a) Role of hippocampal and PFC circuits social memory in encoding and recall

Studies from a wide range of species support the hypothesis that the HPC is essential for both episodic and semantic memory [151]. More recent studies have explored the specific role of the HPC in social memory, which includes neural representations of individuals and social status relationships. Human patients with hippocampal lesions are unable to recognize familiar faces [152,153]. More specifically, studies in rodents have implicated the ventral HPC (vHPC; anterior HPC in primates; aHPC) in modulation of motivated behaviours via connections to the hypothalamus and amygdala, and for its role in valence associative learning [154]. Interconnectivity with the hypothalamus and amygdala and the ability of the vHPC to integrate valence information for memory make this neural system well poised for a role in social memory. In healthy human subjects, neurons in the aHPC are activated when presented with the same individual in a variety of postures and contexts, suggesting that distinct neuronal ensembles represent social memories of individual people [155,156]. Inhibition of the vHPC in mice can impair both memory encoding and recall of individuals, and optogenetically re-activating vHPC neurons that were active during the first social encounter can facilitate these processes [155].

Furthermore, recent studies in mice have identified intra-hippocampal circuits involved in social memory encoding and recall. The HPC consists of CA1, CA2, CA3 and dentate gyrus subregions (figure 2) and extends throughout the medial temporal lobe. Dorsal CA2 (dCA2; posterior in primates) is required to remember littermates in rodents, and projections from dCA2 to ventral CA1 (anterior in primates) encode, store and recall social memories [44,155,157]. Homologous hippocampal structures and subdivisions have been identified in fish, amphibians and reptiles that encode spatial memory similarly to mammals, but little is known about their roles in social memory [99,124,158]. However, in lizards, functional changes in CA3 are observed when status signals are manipulated such that they are incongruous with memory of and opponent's status [99]. Also, in fish, subregions of the lateral part of the dorsal telencephalon (Dl), homologue of the mammalian HPC, exhibit differential *cfos* immunoreactivity under social compared to non-social contexts [124]. Whether the HPC plays a role in learning social ranks in a context of social hierarchies has not been directly tested, however, one study suggests that possibility. BDNF, which enhances neural plasticity required for learning, is upregulated in the HPC of dominant mice after winning agonistic interactions [103], potentially reinforcing their dominant social status.

In mammals, the frontal cortex and its connections to the HPC also facilitate encoding and storage of memories regarding specific group members. Studies in rodents have shown that the vHPC projections to the mPFC are necessary for recalling social memory [159]. Neuroimaging studies in humans indicate that both mPFC and HPC BOLD activity are correlated with learning social ranks in a game [160]. In humans, transcranial current stimulation of rostral mPFC facilitated social rank learning [161]. Furthermore, in mice, mPFC population firing rate is predictive of relative social rank and absolute social rank when competing against conspecifics [92].

In conclusion, there is strong evidence that the role of the HPC in encoding individual social memories is conserved across social species. However, the intricacies of intra-hippocampal circuits and how they facilitate memory formation have predominantly been determined in rodents. In mammals, social memory circuitry extends to the PFC, a region that is critical for long-term memory [151,159]. Although correlative studies in humans point to a role of the HPC–mPFC pathway in social rank learning, this has yet to be demonstrated in animal model studies [160,161]. Further studies into how social memories are stored in the brains of non-mammals are required to determine whether individual recognition is a conserved requirement for stable social hierarchies.

### (b) Neuropeptides support social memory encoding

The neuropeptides OT and arginine vasopressin (AVP) are widely known to regulate social cognition and behaviour across species, with a particular role in supporting social memory. The majority of AVP and OT synthesizing neurons originate in the suprachiasmatic nucleus (SCN) and PVN of the hypothalamus and project widely throughout the brain [162]. One of the major projection targets is the HPC. In rats, hippocampal AVP-receptors are necessary for encoding memory of individual conspecifics [163] and stimulating AVP afferents to dCA2 facilitates social memory encoding [164]. In mice, OTRs in dCA2/CA3 are necessary for short-term [165] and long-term memories of conspecifics [166].

OT and AVP transmission also affect social memory by acting on components of the amygdala and extended amygdala. Studies in mice and rats indicate that the MeA, known for processing social odours, also encodes memories of individual conspecifics. Blocking OTRs in the MeA impairs social recognition in rodents and activating these receptors during an initial encounter enhances their ability to encode social memory [40,167,168]. Also in rodents, the MeA and BNST send AVP projections to the LS, where blockage or downregulation of AVP receptor 1 (V1aR) disrupts social recognition [169]. AVP projections from the MeA to the vHPC have also been confirmed in the rat brain [170], but it is unknown whether these projections are also implicated in social memory.

While evidence from rodents and primates strongly supports the function of neuropeptides in social memory, the function of the homologous neuropeptides in fish and lizards remains to be determined. Arginine vasotocin (AVT), the AVP homologue found in fish and reptiles, has a role in non-social spatial and cue learning [171], but its role in learning in social contexts and about social stimuli has not been established. Notably, AVT has been associated with increased courtship behaviour, aggression and other rank-associated behaviours such as status signalling through urine [172–175]. AVP has been associated with similar social behaviours in mammals [176–178], suggesting that its functions are largely conserved and supporting a case for studying its role in social memory across species. Similarly, isotocin and mesotocin, the OT homologues found in fish and reptiles, respectively, appear to function like OT with respect to social approach, sexual behaviour and formation of partner bonds and parenting behaviour [179–181]. Further investigation into whether AVP and OT homologues modulate memory of conspecifics is needed to know whether these neuropeptides serve a conserved role in social memory encoding across social species.

## 5. Conclusion and future directions

The evidence reviewed supports that social rank recognition involves the coordinated activity of highly conserved neural circuits across multiple levels of cognition, ranging from the seemingly innate perception of social status signals to more fine-tuned learning of social rank of specific individuals. Notably, the amygdala and dopaminergic neurons are involved in responding to status signals and driving learning about social rank through social interactions. While it appears that status signals serve to bypass the need for experience-based learning and prior social interactions that could incur physical injury, the extent to which status signal responses are innate or learned needs to be more thoroughly investigated. This theory, along with several other critical questions about how the brain processes social status signals, needs to be further investigated. In particular, the impact of an animal's familiarity with a social stimulus on their perception of status signals needs to be systematically studied across species. In addition, the role of an animal's own social rank in modulating how they process external status signals is largely unknown. An individual's social rank appears to influence behaviours related to acquiring social information, such as attentional postures and visual gaze direction [3–9], but how social information is differentially represented in the brains of hierarchically ranked animals is understudied. Lastly and perhaps most glaringly absent from our knowledge is how the female brain represents social rank and the neural underpinnings of how females negotiate social rank relationships. Much of the knowledge presented in this review stems from experiments conducted almost exclusively in male animals.

The technical difficulty of studying proximal mechanisms of brain function in naturalistic contexts has been a major hurdle in studying such questions and has led to our limited knowledge of the neural dynamics underlying social group behaviours. Although the species discussed in this review form dominance hierarchies, evidence for the neural systems involved in the representation of social rank typically does not come directly from animals living and behaving freely in groups. Laboratory-based neurobiological and behavioural studies have an overrepresentation of simple dyadic social interaction assays that do not directly examine the representation of social rank in groups, and traditionally measure behaviours that are exclusively expressed by males. Moreover, traditional neural recording methods, such as electrophysiology, have been hard to implement in multiple freely moving animals because of physical constraints. Several recent technological advancements have increased our ability to study the neural basis of social rank learning and memory in larger and more natural group settings. In the past few years, open-source tools have been developed to automatically track and assist in the quantification of behaviour of multiple group-living animals [182–185]. Moreover, technological advancements in light wireless neural activity recording now allow recording from multiple freely moving animals simultaneously [186]. These new developments combined will dramatically facilitate the study of neural circuits and dynamics underlying social group behaviour. We anticipate that the next decade will bring new perspectives on the neurobiology of social group behaviours that will enhance our understanding of how animals in large groups learn and represent social rank.

## Data accessibility

This article has no additional data.
